# Real-World Clinical Outcomes with First-Line Systemic Treatment and Avelumab Maintenance in US Patients with Locally Advanced or Metastatic Urothelial Carcinoma: The SPEAR Bladder-II Study

**DOI:** 10.3390/curroncol32040187

**Published:** 2025-03-24

**Authors:** Sneha Sura, Manojkumar Bupathi, Valerie Morris, Paul Conkling, Karen Todoroff, Abhijeet Bhanegaonkar, Chiemeka Ike

**Affiliations:** 1 Ontada, Boston, MA 02110, USA; sneha.sura@mckesson.com (S.S.); paul.conkling@usoncology.com (P.C.); karen.todoroff@mckesson.com (K.T.); 2 Rocky Mountain Cancer Centers, Littleton, CO 80120, USA; manojkumar.bupathi@usoncology.com; 3 EMD Serono, Inc., Boston, MA 02210, USA, an affiliate of Merck KGaA; valerie.morris@emdserono.com (V.M.); abhijeet.phd@gmail.com (A.B.)

**Keywords:** avelumab, first-line maintenance, locally advanced or metastatic urothelial cancer, overall survival, real-world time to next treatment, real-world time to treatment discontinuation, treatment patterns

## Abstract

Avelumab first-line maintenance (1LM) is approved for patients with locally advanced or metastatic urothelial carcinoma (la/mUC) who do not have disease progression after platinum-based chemotherapy (PBC). This retrospective study describes real-world treatment patterns and clinical outcomes in patients with la/mUC who initiated first-line (1L) systemic treatments, including avelumab 1LM, within iKnowMed, the US community oncology electronic health records database, between 1 December 2019 and 30 November 2023 and followed through 28 February 2024. In total, 1658 patients with la/mUC initiated 1L treatment: immuno-oncology (IO) monotherapy (41.2%), PBC only (32.4%), PBC followed by avelumab 1LM (11.2%), and other treatments (15.1%). The median OS (95% CI) from the start of 1L treatment was 20.4 (13.8, 30.0), 11.0 (8.5, 14.5), and 14.6 (12.6, 17.3) months for cisplatin-based only, carboplatin-based only, and IO monotherapy, respectively. Among the overall population, 36.1% and 11.8% of patients received second-line (2L) and third-line treatment, respectively. The median (95% CI) OS from the start of avelumab 1LM was 18.5 (13.8, 23.8) months. After discontinuation of avelumab 1LM, 43.5% received 2L treatment, and 59.3% of those received enfortumab vedotin (EV); the median (95% CI) OS from start of 2L EV was 12.7 (7.2, 16.5) months. Survival outcomes among patients treated with avelumab 1LM and 2L EV are consistent with respective clinical trials and other real-world studies.

## 1. Introduction

Urothelial carcinoma (UC) is the predominant form of bladder cancer, accounting for 90% of cases [1]. It is the sixth most common malignancy in the United States (US) [1]. Patients with locally advanced or metastatic urothelial cancer (la/mUC) have poor prognosis, with an estimated 5-year survival rate of 8.8% [2]. Platinum-based chemotherapy (PBC) has been the primary treatment for la/mUC for decades. However, recent advancements in targeted therapy and immuno-oncology (IO) agents have transformed the treatment landscape, providing effective options for routine clinical practice [2]. In June 2020, the US Food and Drug Administration (FDA) granted approval to avelumab, a PD-L1 inhibitor, as a first-line maintenance (1LM) treatment for patients with la/mUC whose disease did not progress on PBC. This decision was based on the results from the JAVELIN Bladder 100 phase 3 trial, which demonstrated that avelumab 1LM in combination with best supportive care (BSC) significantly prolonged overall survival (OS) and progression-free survival (PFS) compared to BSC alone [3,4]. As a result, avelumab 1LM has been established as a recommended treatment option for patients with la/mUC whose disease has not progressed following 1L PBC according to the National Comprehensive Cancer Network (NCCN) guidelines [5].

Moreover, recent updates to clinical guidelines have included additional first-line combination treatment options for patients with la/mUC [5,6]. The combination of enfortumab vedotin (EV) and pembrolizumab initially received accelerated approval in April 2023 for cisplatin-ineligible patients based on the EV-103 trial [7,8] and subsequently received full FDA approval in December 2023, regardless of cisplatin eligibility, based on the phase 3 EV-302 trial, which demonstrated significant improvements in PFS and OS compared to PBC alone [9]. Additionally, for cisplatin-eligible patients, the combination of nivolumab, gemcitabine, and cisplatin followed by nivolumab maintenance received FDA approval in March 2024, based on the phase 3 CheckMate-901 trial sub-study, which showed significant improvements in PFS and OS compared to cisplatin–gemcitabine alone [10]. IO monotherapy, including pembrolizumab and atezolizumab monotherapies, are options for 1L treatment of patients who are ineligible for any platinum-based treatment [1].

While these advancements have greatly expanded 1L treatment options for patients with la/mUC, there remain questions regarding the optimal sequencing of newly available treatment options with effective therapies in second-line (2L) and beyond settings for patients who are refractory to PBC and IO. The NCCN guidelines recommend a variety of subsequent treatment options depending on the prior line of therapy [5]. Pembrolizumab monotherapy is preferred after progression on 1L PBC based on the KEYNOTE-045 study [11]. EV, an antibody–drug conjugate (ADC) targeting Nectin-4, is preferred after 1L PBC and IO treatment based on the phase 3 EV-301 study [12]. For patients with susceptible *FGFR3* genetic alterations, the FGFR inhibitor erdafitinib received FDA approval based on the phase 3 THOR study [13]. In addition, the ADC targeting Trop-2, sacituzumab govitecan (SG), was recommended as a subsequent therapy with accelerated approval based on the phase 2 TROPHY-U-01 trial; however, results from the TROPiCS-04 phase 3 trial led to the manufacturer voluntarily withdrawing this indication [14,15,16]. Other options for subsequent treatment include PBC rechallenge or single-agent or combination nonplatinum chemotherapies (e.g., taxanes) [5].

Despite the availability of these novel treatment options, there is a need to understand the real-world utilization, treatment patterns, and clinical outcomes with current 1L and subsequent systemic therapies, including avelumab 1LM, in patients with la/mUC in the US community oncology setting. Evaluating real-world data provides valuable insights into treatment sequence approaches and the effectiveness of avelumab 1LM in routine clinical practice, complementing the evidence obtained from clinical trials. This study aimed to evaluate treatment patterns and clinical outcomes among patients with la/mUC initiating 1L treatments within the US community oncology setting post FDA approval of avelumab 1LM treatment. In addition, we evaluated the real-world use and clinical outcomes of EV after avelumab 1LM progression, which was not approved at the time of the pivotal clinical trial JAVELIN Bladder 100.

## 2. Materials and Methods

### 2.1. Data Source

We utilized The US Oncology Network’s system, iKnowMed (iKM), and its electronic health record (EHR) data. The US Oncology Network treats over 1.2 million patients annually by 1400 affiliated physicians operating in more than 500 geographically diverse community-based outpatient practices across the US [7]. Study data were sourced from the structured field of the iKM EHR database, with supplementary vital status obtained from commercially available datasets that contain death dates sourced from repositories of claims data, obituary records, and the Social Security Administration’s Death Master File (LADMF). This study was granted an exception and waiver of consent by The US Oncology Inc. Institutional Review Board.

### 2.2. Study Design and Population

This retrospective observational cohort study included adult (≥18 years) patients with a diagnosis of la/mUC who initiated 1L systemic anticancer treatments within The US Oncology Network between 1 December 2019 and 30 November 2023 and were followed longitudinally through the end of the study observation period (28 February 2024), the date of the last visit, or the date of death, whichever occurred first. The date of initiation of 1L systemic treatments following diagnosis of la/mUC was defined as the index date. Eligible patients were required to have a minimum of two physician visits with The US Oncology Network during the study’s observation period. Patients enrolled in interventional clinical trials or those who received treatment for another primary cancer diagnosis besides la/mUC during the study period were excluded.

### 2.3. Study Measures

#### 2.3.1. Patient Characteristics

Patient characteristics were assessed during a period of 60 days before the index date, which included gender, race, and practice location. Clinical characteristics included muscle invasion, metastatic sites, Eastern Cooperative Oncology Group (ECOG) Performance Status (PS), primary tumor location, and comorbidities. Age was captured at the index date.

#### 2.3.2. Line of Treatment Algorithm and Sequencing

Treatment patterns were characterized using a line of treatment (LOT) algorithm and included the distribution and sequence of regimens received in the advanced/metastatic setting. The first systemic treatment received after the initial la/mUC diagnosis was considered 1L. Drugs administered within 28 days of the current treatment were considered a combination therapy. New treatments seen 28 days after the current therapy or with a gap of greater than 90 days were considered the next LOT. If avelumab was seen within 90 days of completion of the 1L PBC, the avelumab regimen was considered maintenance treatment.

#### 2.3.3. Clinical Outcomes 

We examined clinical effectiveness outcomes, such as OS, real-world time to treatment discontinuation (rwTTD), and real-world time to next treatment (rwTTNT). Patients were followed from the index date until the end of the study’s observation period; patients without the event of interest on their last contact date prior to the end of the study’s observation period were censored. OS was defined as the interval between the index date and the date of death by any cause. rwTTD was defined as the interval between treatment initiation and treatment discontinuation, including any treatment interruptions or other breaks (no more than 60 consecutive days in length). rwTTD did not include any clinical benefit days derived from the last dose. Treatment discontinuation for infused treatments was observed on the last administration date of infused treatments. Patients without a second infused treatment administration had a duration of 1 day. For oral therapies, if the date of discontinuation was not recorded, the date of discontinuation was inferred to be the date of death, the date of treatment switch or the addition of another anticancer regimen, or the last prescription date if the treatment occurred ≥120 days before the last contact date, whichever occurred first. rwTTNT was defined as the interval between the index date and initiation of the next treatment or the date of death due to any cause. Among patients who received avelumab 1LM, outcomes were evaluated from the start of avelumab 1LM. Among patients who received 2L EV post avelumab 1LM, outcomes were evaluated from the start of 2L EV.

### 2.4. Statistical Analysis

Patient demographic and clinical characteristics were summarized for the overall cohort and by 1L systemic treatments. Categorical variables were described using counts and percentages. Continuous variables were summarized using the mean, the standard deviation (SD), the median, the minimum, and the maximum. Treatment patterns were summarized descriptively using patient counts and percentages. Time-to-event outcomes were estimated using the Kaplan–Meier method. Median survival with 95% confidence intervals (CIs) and survival probabilities (with 95% CIs) were assessed. Cell counts with fewer than five patients were suppressed due to data privacy regulations. All analyses were conducted using SAS 9.4.

## 3. Results

### 3.1. Study Population

We identified a total of 1658 patients with la/mUC who initiated 1L systemic treatment (Figure S1), of whom 683 (41.2%) received IO monotherapies, 305 (18.4%) received cisplatin-based only, 233 (14.1%) received carboplatin-based only, 147 (8.9%) received ADC, 93 (5.6%) received cisplatin with avelumab 1LM, 93 (5.6%) received carboplatin with avelumab 1LM, 24 (1.4%) received avelumab monotherapy, and 80 (4.8%) received other therapies.

### 3.2. Demographics and Clinical Characteristics

Overall, the median (min, max) age at initiation of 1L systemic treatments was 73 (31, 90+) years (Table 1); furthermore, 74.7% were male and 73.3% were Caucasian. Most (47.1%) patients had an ECOG PS of ≤1 at baseline. The median (range) follow-up time from the index date was 9.0 (0.1, 50.4) months. Across 1L treatments, the median age ranged from 67 years (cisplatin-based only/cisplatin with avelumab 1LM) to 78 years (IO monotherapy). The median follow-up time ranged from 5.2 months (ADC) to 12.7 months (carboplatin with avelumab 1LM; Table 1). The median (range) follow-up from the start of avelumab 1LM was 9.1 (0.5–42.2) months.

### 3.3. 1L Treatment Patterns

**Figure 1:**
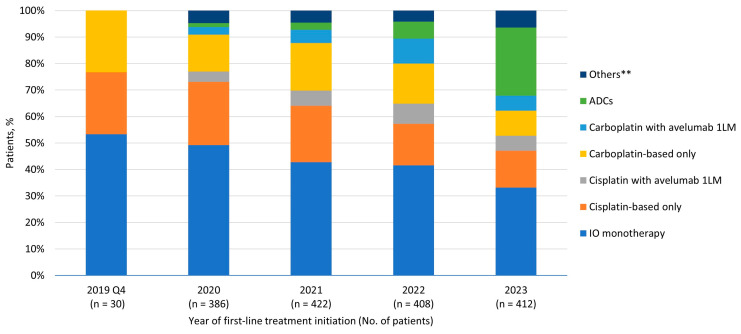
1L systemic treatment trends by year among patients with la/mUC initiating 1L treatments. Abbreviations: 1L—first-line; 1LM—first-line maintenance; ADC—antibody-drug conjugate; IO—immuno-oncology; la/mUC—locally advanced or metastatic urothelial carcinoma. ** Other treatments included gemcitabine, erdafitinib, fluorouracil, capecitabine, and methotrexate, as monotherapy or in combination.

Pembrolizumab (n = 558, 81.7%) and atezolizumab (n = 89, 13.0%) were the most common 1L treatments among patients receiving IO monotherapy (n = 683). Among patients receiving PBC (n = 724), cisplatin–gemcitabine (n = 337, 46.5%) and carboplatin–gemcitabine (n = 272, 37.6%) were the most common treatments, and, in total, 186 (25.7%) received avelumab 1LM post PBC. Among patients receiving ADC (n = 147), EV plus pembrolizumab (n = 87, 59.2%) and EV monotherapy (n = 52, 35.4%) were the most common treatments. Among patients receiving other treatments (n = 80), gemcitabine monotherapy (n = 22, 27.5%) and fluorouracil monotherapy (n = 19, 23.8%) were the top treatments. Trends of 1L systemic treatment by year are shown in Figure 1.

**Figure 2:**
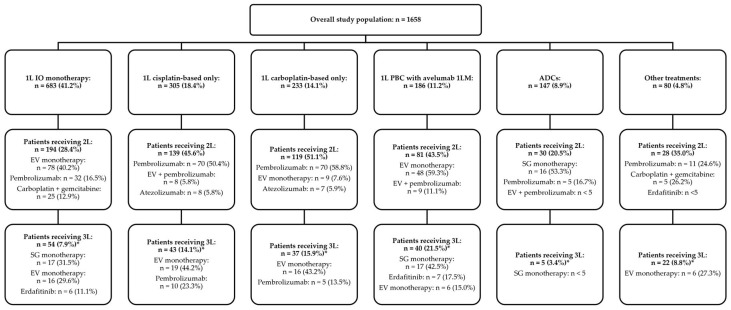
Treatment patterns among patients with la/mUC initiating 1L systemic treatments. Abbreviations: 1L—first-line; 1LM—first-line maintenance; 2L—second-line; 3L—third-line; ADC—antibody-drug conjugate; EV—regimens containing enfortumab vedotin; IO—immuno-oncology; la/mUC—locally advanced or metastatic urothelial carcinoma; PBC—platinum-based chemotherapy; SG—sacituzumab govitecan. Other treatments included other chemotherapy, IO–chemotherapy combination, and non-chemotherapy/non-IO treatments. The treatment patterns include up to 3 of the most common treatments for each line of therapy. * Percentages are calculated out of patients receiving 1L treatments.

Among the overall study cohort who received 1L treatments (n = 1658), 598 (36.1%) and 196 (11.8%) patients received 2L and third-line (3L) treatments, respectively. The most common 2L treatments were pembrolizumab monotherapy (n = 194, 32.4%), EV monotherapy (n = 143, 23.9%), and EV plus pembrolizumab (n = 44, 7.4%). The most common 3L treatments were EV monotherapy (n = 63, 32.1%), SG (n = 39, 19.9%), and pembrolizumab monotherapy (n = 25, 12.8%). During the study observation period, 44 (23.7%) patients remained on avelumab 1LM, and 81 (43.5%) patients received 2L treatments after avelumab 1LM and 1L PBC; furthermore, 48 (59.3%) of those received 2L EV monotherapy (Figure 2).

### 3.4. Clinical Outcomes

#### 3.4.1. Overall Survival (OS) 

The median (95% CI) OS from the start of the index date was 20.4 (13.8, 30.0) months for cisplatin-based treatments only, 11.0 (8.5, 14.5) months for carboplatin-based treatments only, and 14.6 (12.6, 17.3) months for IO monotherapy (Table 2 and Figure S2A). Among patients who received avelumab 1LM, the median OS from avelumab 1LM initiation was 18.5 (13.8, 23.8) months (Table 3 and Figure S3A). Median OS from initiation of 2L EV was 12.7 (7.2, 16.5) months among patients who received avelumab 1LM (Table 3 and Figure S4A).

#### 3.4.2. Real-World Time to Treatment Discontinuation (rwTTD) 

Across 1L treatments, the median rwTTD (95% CI) from the start of index date was 2.3 (2.1, 2.3) months for cisplatin-based treatments only, 2.1 (1.9, 2.3) months for carboplatin-based treatments only, and 3.7 (3.4, 4.2) months for IO monotherapy (Table 2 and Figure S2B). Among patients who received avelumab 1LM, the median rwTTD from the start of avelumab 1LM was 4.6 (3.5, 5.6) months (Table 3 and Figure S3B). The median rwTTD from initiation of 2L EV was 4.6 (2.4, 6.7) months among those who received avelumab 1LM (Table 3 and Figure S4B).

#### 3.4.3. Real-World Time to Next Treatment (rwTTNT) 

Across 1L treatments, the median rwTTNT (95% CI) from the start of the index date was 6.5 (5.9, 7.2) months for cisplatin-based treatments only, 4.7 (3.9, 5.4) months for carboplatin-based treatments only, and 8.0 (7.3, 9.1) months for IO monotherapy (Table 2 and Figure S2C). Among patients initiating avelumab 1LM, the median rwTTNT from the start of avelumab 1LM was 6.5 (5.6, 7.2) months (Table 3 and Figure S3C). The median rwTTNT from initiation of 2L EV was 6.1 (4.6, 7.9) months among those who received avelumab 1LM (Table 3 and Figure S4C).

## 4. Discussion

This real-world study evaluated the effectiveness of avelumab 1LM among other contemporary 1L and 2L treatment patterns in the evolving la/mUC landscape in a large US community oncology setting. Treatment patterns observed in this study are consistent with the NCCN guidelines at the time of the study [5]. Of patients receiving 1L systemic treatment, 43.7% received 1L PBC and 41.2% received IO monotherapy. This high proportion of IO monotherapy use was observed in other real-world studies capturing contemporary treatments in the US community setting [17,18,19]. After the FDA approval of avelumab 1LM in June 2020 [3], we noted an increase in the uptake of avelumab 1LM over time, from 25.0% in 2020 to 32.9% in 2023. The increased use of avelumab 1LM may be attributed to greater awareness among physicians and patient preference, indicating that further education on its benefits may be needed for community clinicians. Previous studies have shown similar uptake rates of avelumab 1LM in real-world US community oncology settings. For instance, an analysis based on Flatiron data in patients with advanced UC indicated that 20% of the 1L PBC treated patients received avelumab 1LM [20]. Another study employing iKM EHR data found that 32% of the 1L PBC treated patients received avelumab 1LM [17]. We observed recent uptake of ADCs alone or in combination in the 1L setting among 8.9% of the overall cohort, 59.2% of whom received EV plus pembrolizumab, which was initially approved in April 2023 for the cisplatin-ineligible la/mUC population [7,8].

Clinical outcomes among patients who received avelumab 1LM were similar to those in previous clinical trials and real-world studies; however, median follow-up times varied across these studies. The median OS from the start of avelumab 1LM (18.5 months) in our study was slightly lower than that observed in the JAVELIN Bladder 100 trial (21.4 months and 23.8 months) [3,4] and two US real-world studies (20.6 and 23.8 months) [21,22]. Furthermore, the US PATRIOT-II retrospective chart review study reported a median OS of 24.4 months [23]. The median rwTTD and rwTTNT from start of avelumab 1LM (4.6 and 6.5 months) observed in our study were comparable to prior real-world US studies (3.9 and 7.0 months) [21,24]. Previous clinical trials and other real-world studies reported progression-free survival (PFS), but not TTD and TNNT.

When the JAVELIN Bladder 100 trial was conducted, contemporary 2L treatments of erdafitinib, EV, and SG were not available. This study examined the treatment patterns and outcomes in particular for 2L EV in patients who discontinued avelumab 1LM post 1L PBC. Median rwTTD (4.6 months) and rwTTNT (6.5 months) from the start of 2L EV observed in this study was similar to median PFS from the EV-301 clinical trial (5.6 months) [25]. Similar findings were observed in other real-world studies (median PFS 4.9–6.6 months) [21,22,26]. The median OS from the start of 2L EV in our study was 12.7 months, which is consistent with the results of the EV-301 clinical trial (12.9 months) [25] and in line with the median OS reported in other real-world studies (11.2–14.5 months) [21,22,26,27].

This study captured clinical outcomes for other available 1L treatment options for patients with la/mUC during the same time period. Patients who received cisplatin-based chemotherapy only (without avelumab 1LM) had a median OS of 20.4 months, whereas patients who received IO monotherapy or carboplatin-based chemotherapy only had a median OS of 14.6 or 11.0 months, respectively. While reported for the 1L ADC cohort, the median OS of 9.6 months should be interpreted with caution, as it included EV as both a monotherapy and in combination with pembrolizumab and has a shorter follow-up time with a median of only 5.2 months. Further evaluation of real-world clinical outcomes for patients with la/mUC treated with recently approved 1L treatments, such as EV plus pembrolizumab and nivolumab, gemcitabine, and cisplatin followed by nivolumab maintenance, is needed [5,9,10].

The high attrition rates after LOTs indicated an unmet need for patients with la/mUC. Consistent with previous studies, our study found that nearly two-thirds of patients did not receive 2L and nearly nine out of ten did not receive 3L treatments [28,29,30]. Galsky et al. (2018), using the Surveillance, Epidemiology, and End Results (SEER)-Medicare database, reported that only 35% of patients with la/mUC who initiated 1L proceeded to a 2L treatment [31]. A systematic review and meta-analysis of 47 studies reported the rate of subsequent LOTs with the median proportion of patients advancing from 1L to 2L and from 2L to 3L treatment as 43% (range 8–87%) and 30% (range 8–77%), respectively [29]. The approval of IO monotherapies and targeted treatments, such as erdafitinib and ADCs, for the treatment of patients with la/mUC has provided some additional 2L treatment options for physicians [13,14]. Furthermore, the choice of 2L treatment often depends on the 1L treatment received [5]. In our study, over half of the patients received EV monotherapy post 1L IO monotherapy, which is consistent with other real-world studies [21,22].

Because this analysis was based on retrospective, nonrandomized data, these results should be considered in context with clinical trial and other real-world data. The iKM EHR database is used for clinical practice purposes. Thus, physician reporting practices could introduce selection bias and affect data standardization and collection methods. Consequently, the use of structured data only may introduce some level of misclassification bias due to data entry errors or missing values. Moreover, the absence of tumor response may result in misclassification between 2L avelumab and 1LM avelumab. Services and procedures provided outside of physician offices (e.g., hospitalizations) are not captured by the database. Oral therapies like erdafitinib were recorded as prescribed through iKM, but fulfillment of those prescriptions was not observable. Practices within The US Oncology Network may have patient populations and/or prescribing practices that differ from other community oncology clinics, which may limit generalizability to other oncology settings and may not be generalizable to academic practices. The study also has a limited follow-up of 9 months for the overall cohort; longer follow-up for assessing any clinical outcomes is warranted.

## 5. Conclusions

These real-world study findings demonstrate avelumab 1LM’s effectiveness for patients whose disease has not progressed on 1L PBC and provide evidence for the use of 2L EV after avelumab 1LM. There was an increased uptake of avelumab 1LM since its FDA approval. Survival outcomes are consistent with the JAVELIN Bladder 100 clinical trial and other real-world studies that had longer follow-up times. While EV plus pembrolizumab is the preferred 1L therapy in updated clinical guidelines [5], PBC followed by avelumab 1LM remains a recommended treatment option for patients who may not be suitable for EV plus pembrolizumab. Future studies with longer follow-up are needed to further characterize clinical outcomes in non-academic settings to optimize treatment selection and inform sequencing in patients with la/mUC considering the recent approval of novel therapies in the US.

## Figures and Tables

**Table 1:** Demographic and clinical characteristics of patients with la/mUC initiating 1L systemic treatments.

Characteristics	Overall * (n = 1658)	IO Monotherapy (n = 683)	Cisplatin-Based Only (n = 305)	Carboplatin-Based Only (n = 233)	Cisplatin with Avelumab 1LM (n = 93)	Carboplatin with Avelumab 1LM (n = 93)	ADCs (n = 147)	Others ** (n = 80)
**Age at diagnosis (years)**								
Mean (SD)	72.7 (10.1)	76.4 (9.9)	67.1 (8.7)	72.5 (9.3)	66.2 (8.3)	73.8 (7.3)	71.7 (9.8)	72.0 (10.7)
Median (Min, Max)	73 (31, 90+)	78 (41, 90+)	67 (40, 87)	74 (31, 90+)	67 (47, 84)	74 (50, 89)	73 (49, 90+)	72 (40, 90+)
**Gender, n (%)**								
Female	420 (25.3)	186 (27.2)	59 (19.3)	60 (25.8)	26 (28.0)	18 (19.4)	35 (23.8)	26 (32.5)
Male	1238 (74.7)	497 (72.8)	246 (80.7)	173 (74.2)	67 (72.0)	75 (80.6)	112 (76.2)	54 (67.5)
**Race, n (%)**								
White or Caucasian	1216 (73.3)	506 (74.1)	223 (73.1)	171 (73.4)	74 (79.6)	71 (76.3)	96 (65.3)	58 (72.5)
Other	192 (11.6)	71 (10.4)	37 (12.1)	28 (12.0)	11 (11.8)	8 (8.6)	18 (12.2)	15 (18.8)
Not documented	250 (15.1)	106 (15.5)	45 (14.8)	34 (14.6)	8 (8.6)	14 (15.1)	33 (22.4)	7 (8.8)
**BMI (kg/m^2^)**								
Mean (SD)	26.9 (5.4)	26.4 (5.2)	27.5 (5.3)	27.2 (5.5)	27.3 (6.0)	27.8 (4.8)	26.7 (5.4)	27.4 (6.0)
Median (Min, Max)	26.4 (14.4, 46.6)	25.8 (14.4, 46.6)	27.1 (16.4, 44.2)	26.6 (17.1, 44.2)	26.3 (17.0, 42.7)	27.1 (17.5, 43.5)	26.3 (15.4, 46.0)	27.5 (17.1, 46.1)
**Tobacco Use, n (%)**								
No history of tobacco use	279 (16.8)	115 (16.8)	58 (19.0)	34 (14.6)	15 (16.1)	20 (21.5)	19 (12.9)	13 (16.3)
Current tobacco use	136 (8.2)	39 (5.7)	43 (14.1)	25 (10.7)	9 (9.7)	<5	7 (4.8)	7 (8.8)
Former tobacco use	435 (26.2)	159 (23.3)	98 (32.1)	61 (26.2)	33 (35.5)	29 (31.2)	28 (19.0)	23 (28.8)
No information	808 (48.7)	370 (54.2)	106 (34.8)	113 (48.5)	36 (38.7)	42 (45.2)	93 (63.3)	37 (46.3)
**ECOG PS, n (%)**								
0	213 (12.8)	81 (11.9)	56 (18.4)	27 (11.6)	19 (20.4)	7 (7.5)	14 (9.5)	8 (10.0)
1	568 (34.3)	232 (34.0)	93 (30.5)	78 (33.5)	41 (44.1)	37 (39.8)	44 (29.9)	31 (38.8)
2	134 (8.1)	75 (11.0)	9 (3.0)	22 (9.4)	<5	<5	15 (10.2)	6 (7.5)
3+	23 (1.4)	17 (2.5)	<5	<5	<5	0 (0.0)	0 (0.0)	0 (0.0)
No information	720 (43.4)	278 (40.7)	146 (47.9)	102 (43.8)	30 (32.3)	45 (48.4)	74 (50.3)	35 (43.8)
**Distant metastatic sites, n (%)**								
Bone	79 (4.8)	19 (2.8)	17 (5.6)	17 (7.3)	9 (9.7)	5 (5.4)	5 (3.4)	5 (6.3)
Brain	5 (0.3)	<5	<5	<5	0 (0.0)	0 (0.0)	0 (0.0)	0 (0.0)
Liver	29 (1.7)	7 (1.0)	7 (2.3)	6 (2.6)	<5	<5	<5	<5
Lung	54 (3.3)	25 (3.7)	8 (2.6)	5 (2.1)	<5	5 (5.4)	5 (3.4)	<5
Other	89 (5.4)	34 (5.0)	13 (4.3)	15 (6.4)	9 (9.7)	5 (5.4)	8 (5.4)	<5
**Index year, n (%)**								
2019 Q4	30 (1.8)	16 (2.3)	7 (2.3)	7 (3.0)	0 (0.0)	0 (0.0)	0 (0.0)	0 (0.0)
2020	386 (23.3)	188 (27.5)	91 (29.8)	53 (22.7)	15 (16.1)	11 (11.8)	6 (4.1)	18 (22.5)
2021	422 (25.5)	177 (25.9)	88 (28.9)	74 (31.8)	24 (25.8)	21 (22.6)	11 (7.5)	19 (23.8)
2022	408 (24.6)	168 (24.6)	63 (20.7)	61 (26.2)	31 (33.3)	38 (40.9)	26 (17.7)	17 (21.3)
2023	412 (24.8)	134 (19.6)	56 (18.4)	38 (16.3)	23 (24.7)	23 (24.7)	104 (70.7)	26 (32.5)
**Follow-up duration (months)**								
Mean (SD)	12.7 (10.9)	13.0 (11.1)	14.1 (12.3)	12.7 (11.7)	15.2 (9.4)	15.3 (9.1)	6.6 (5.8)	11.9 (10.1)
Median (Min, Max)	9.0 (0.1, 50.4)	9.4 (0.7, 49.2)	9.5 (0.1, 49.0)	8.2 (0.3, 50.4)	12.2 (3.1, 41.0)	12.7 (2.1, 45.1)	5.2 (0.5, 34.7)	8.2 (1.0, 43.7)

Abbreviations: 1L—first-line; 1LM—first-line maintenance; ADC—antibody-drug conjugate; BMI—body mass index; ECOG—Eastern Cooperative Oncology Group; IO—immuno-oncology; la/mUC—locally advanced or metastatic urothelial carcinoma; Max—maximum; Min—minimum; PS—performance score; SD—standard deviation. * Patient characteristics are not reported for patients initiating 1L avelumab monotherapy due to the small sample size (n = 24). ** Other treatments included gemcitabine, erdafitinib, fluorouracil, capecitabine, and methotrexate, as monotherapy or in combination.

**Table 2:** Clinical outcomes among patients with la/mUC initiating 1L systemic treatments.

Clinical Outcomes *	Overall ** (n = 1658)	IO Monotherapy (n = 683)	Cisplatin-Based Only (n = 305)	Carboplatin-Based Only (n = 233)	ADCs (n = 147)	Others *** (n = 80)
**Overall survival (OS)**						
Events, n (%)	854 (52.3)	392 (57.4)	140 (45.9)	146 (62.7)	60 (40.8)	34 (42.5)
Median, months (95% CI)	15.3 (13.8, 17.0)	14.6 (12.6, 17.3)	20.4 (13.8, 30.0)	11.0 (8.5, 14.5)	9.6 (8.0, 11.9)	17.3 (12.1, 32.1)
OS probability, % (95% CI)						
At 6 months	76.1% (73.8, 78.1)	72.6% (69.0, 75.9)	81.8% (76.7, 85.8)	67.6% (61.1, 73.3)	72.3% (63.2, 79.6)	76.3% (64.6, 84.6)
At 12 months	56.5% (53.9, 59.1)	55.0% (50.9, 58.9)	60.7% (54.4, 66.5)	47.3% (40.4, 53.8)	36.2% (24.0, 48.5)	66.5% (53.2, 76.8)
At 18 months	45.5% (42.7, 48.3)	44.8% (40.5, 48.9)	50.2% (43.5, 56.5)	35.1% (28.4, 41.8)	32.6% (20.1, 45.7)	48.3% (34.1, 61.2)
At 24 months	37.9% (34.9, 40.8)	35.4% (31.1, 39.8)	46.7% (39.8, 53.2)	29.4% (22.9, 36.2)	26.1% (12.3, 42.1)	40.8% (25.8, 55.3)
**Real-world time to treatment discontinuation (rwTTD)**						
Events, n (%)	1414 (86.5)	589 (86.2)	291 (95.4)	224 (96.1)	95 (64.6)	73 (91.3)
Median, months (95% CI)	3.0 (2.8, 3.4)	3.7 (3.4, 4.2)	2.3 (2.1, 2.3)	2.1 (1.9, 2.3)	4.4 (3.0, 5.3)	1.0 (0.8, 1.5)
rwTTD probability, % (95% CI)						
At 6 months	30.5% (28.2, 32.8)	38.1% (34.5, 41.8)	3.7% (1.9, 6.5)	9.0% (5.7, 13.1)	38.5% (29.9, 47.0)	17.3% (9.8, 26.6)
At 12 months	15.1% (13.3, 17.1)	20.6% (17.5, 23.9)	1.0% (0.1, 4.0)	4.2% (2.0, 7.4)	20.8% (11.9, 31.4)	9.2% (3.7, 17.8)
At 18 months	9.4% (7.8, 11.2)	13.5% (10.8, 16.6)	NA	3.1% (1.3, 6.2)	15.6% (6.3, 28.7)	4.6% (1.0, 12.9)
At 24 months	6.4% (5.0, 8.0)	8.9% (6.5, 11.7)	NA	3.1% (1.3, 6.2)	15.6% (6.3, 28.7)	NA
**Real-world time to next treatment (rwTTNT)**						
Events, n (%)	1128 (69.0)	469 (68.7)	209 (68.5)	198 (85.0)	75 (51.0)	49 (61.3)
Median, months (95% CI)	7.4 (6.9, 7.8)	8.0 (7.3, 9.1)	6.5 (5.9, 7.2)	4.7 (3.9, 5.4)	6.4 (5.8, 7.6)	8.0 (6.7, 11.8)
rwTTNT probability, % (95% CI)						
At 6 months	59.4% (56.9, 61.8)	60.1% (56.2, 63.8)	55.3% (49.2, 61.0)	39.2% (32.8, 45.5)	58.6% (48.9, 67.1)	62.2% (50.1, 72.2)
At 12 months	32.7% (30.2, 35.2)	37.6% (33.8, 41.5)	28.0% (22.5, 33.7)	18.5% (13.6, 24.0)	26.7% (16.3, 38.2)	35.8% (23.6, 48.1)
At 18 months	25.2% (22.7, 27.6)	30.7% (26.9, 34.6)	23.6% (18.4, 29.2)	13.2% (8.9, 18.2)	16.9% (7.5, 29.6)	26.1% (14.9, 38.8)
At 24 months	20.0% (17.7, 22.5)	24.3% (20.5, 28.2)	21.6% (16.4, 27.2)	12.0% (8.0, 17.0)	11.3% (3.0, 25.9)	11.6% (2.8, 27.2)

Abbreviations: 1L—first-line; 1LM—first-line maintenance; IO—immuno-oncology; la/mUC—locally advanced or metastatic urothelial carcinoma; NA—not applicable. * Clinical outcomes are not reported for patients initiating 1L avelumab due to small sample size (n = 24). ** Clinical outcomes were calculated from start of 1L systemic treatments. *** Other treatments included gemcitabine, erdafitinib, fluorouracil, capecitabine, and methotrexate, as monotherapy or in combination.

**Table 3:** Clinical outcomes among patients with la/mUC initiating avelumab 1LM and subsequent 2L EV.

Clinical Outcomes	Avelumab 1LM * (n = 186)	2L EV Post Avelumab 1LM ** (n = 57)
**Overall survival (OS)**		
Events, n (%)	82 (44.1)	28 (49.1)
Median, months (95% CI)	18.5 (13.8, 23.8)	12.7 (7.2, 16.5)
OS probability, % (95% CI)		
At 6 months	77.5% (70.4, 83.1)	77.7% (63.1, 87.1)
At 12 months	61.2% (52.8, 68.6)	51.1% (33.8, 66.0)
At 18 months	51.2% (42.1, 59.5)	20.2% (7.1, 37.9)
At 24 months	38.2% (27.9, 48.3)	NA
**Real-world time to treatment discontinuation (rwTTD)**		
Events, n (%)	142 (76.3)	40 (70.2)
Median, months (95% CI)	4.6 (3.5, 5.6)	4.6 (2.4, 6.7)
rwTTD probability, % (95% CI)		
At 6 months	38.3% (31.0, 45.6)	38.5% (24.9, 52.0)
At 12 months	19.0% (13.1, 25.7)	11.4% (3.2, 25.3)
At 18 months	13.8% (8.4, 20.5)	5.7% (0.5, 20.6)
At 24 months	11.0% (6.0, 17.8)	NA
**Real-world time to next treatment (rwTTNT)**		
Events, n (%)	128 (68.8)	38 (66.7)
Median, months (95% CI)	6.5 (5.6, 7.2)	6.1 (4.6, 7.9)
rwTTNT probability, % (95% CI)		
At 6 months	52.4% (44.5, 59.7)	52.7% (37.6, 65.8)
At 12 months	26.0% (19.1, 33.4)	10.0% (2.7, 23.1)
At 18 months	18.5% (12.1, 25.9)	5.0% (0.5, 18.6)
At 24 months	14.7% (8.7, 22.1)	NA

Abbreviations: 1L—first-line; 1LM—first-line maintenance; 2L—second-line; EV—enfortumab vedotin; la/mUC—locally advanced or metastatic urothelial carcinoma; NA—not applicable. * Clinical outcomes were calculated from start of avelumab 1LM in patients without progression on 1L PBC. ** Clinical outcomes were calculated from start of 2L EV.

## Data Availability

Per our IRB exempt determination/waiver of authorization and consent, all patient-level data are de-identified in accordance with HIPAA Sections 164.514(b) and (c). The data that support the findings of this study are available from Ontada, a McKesson Corporation business, but restrictions apply to the availability of these data due to the risk of reidentification. We are unable to share these data outside of the organization.

## Supplementary Materials

The following supporting information can be downloaded at: https://www.mdpi.com/article/10.3390/curroncol32040187/s1, Figure S1: Selection criteria of patients with la/mUC; Figure S2: Clinical outcomes among patients with la/mUC initiating 1L systemic treatments without avelumab 1LM; Figure S3: Clinical outcomes among patients with la/mUC initiating avelumab 1LM post 1L PBC; Figure S4: Clinical outcomes among patients with la/mUC initiating 2L EV post avelumab 1LM.
