# Supplementary material for: Real-World Clinical Outcomes with First-Line Systemic Treatment and Avelumab Maintenance in US Patients with Locally Advanced or Metastatic Urothelial Carcinoma: The SPEAR Bladder-II Study
Source: Curr Oncol. 2025 Mar 24;32(4):187. doi: 10.3390/curroncol32040187 (PMC12025360; doi:10.3390/curroncol32040187)
Supplement: Supplementary file 1 [file curroncol-32-00187-s001.zip › curroncol-3410608-supplementary.pdf]

Supplementary Figures

Figure S1. Selection criteria of patients with la/mUC

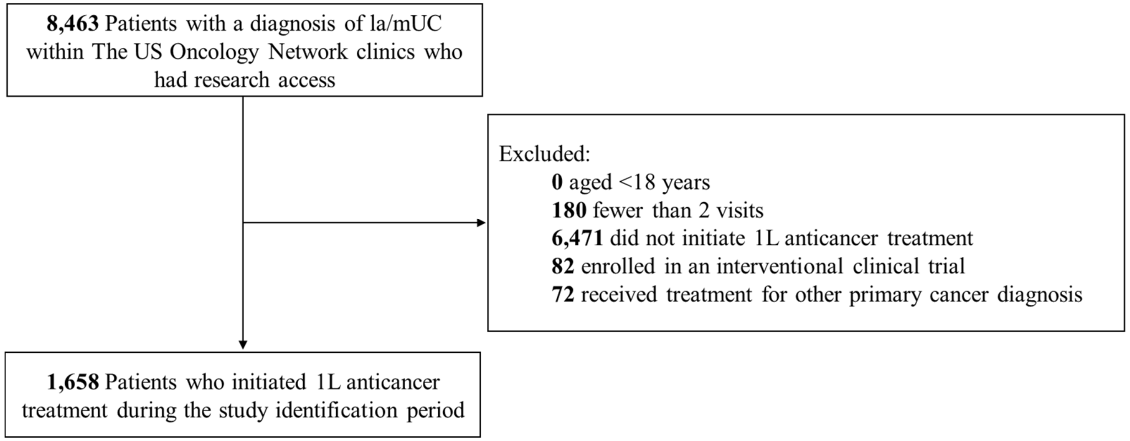

Abbreviations: 1L - first line; la/mUC - locally advanced or metastatic urothelial carcinoma

Figure S2. Clinical outcomes among patients with la/mUC initiating 1L systemic treatments without avelumab 1LM

A. Overall survival (OS)

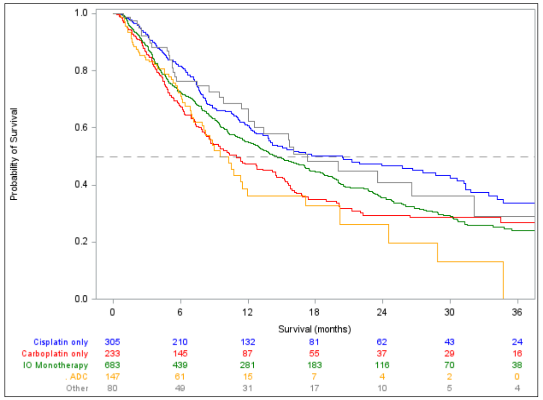

B. Real-world time to treatment discontinuation (rwTTD)

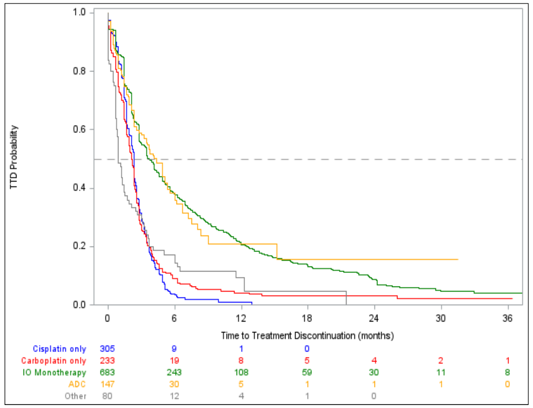

C. Real-world time to next treatment (rwTTNT)

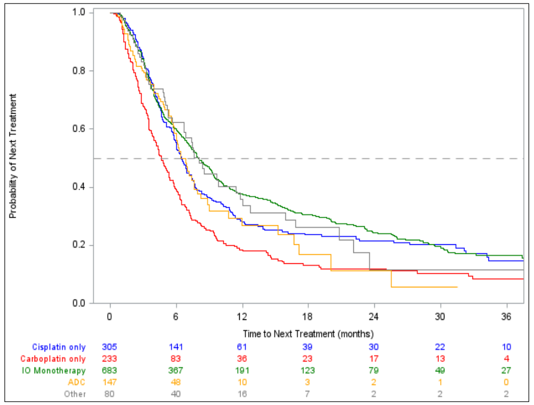

Abbreviations: 1L - first line; 1LM - first-line maintenance; ADC - antibody drug conjugate; IO - immuno-oncology; la/mUC - locally advanced or metastatic urothelial carcinoma

Clinical outcomes were calculated from start of 1L systemic treatments (index date)

Figure S3. Clinical outcomes among patients with la/mUC initiating avelumab 1LM post 1L PBC

A. Overall survival (OS)

B. Real-world time to treatment discontinuation (rwTTD)

C. Real-world time to next treatment (rwTTNT)

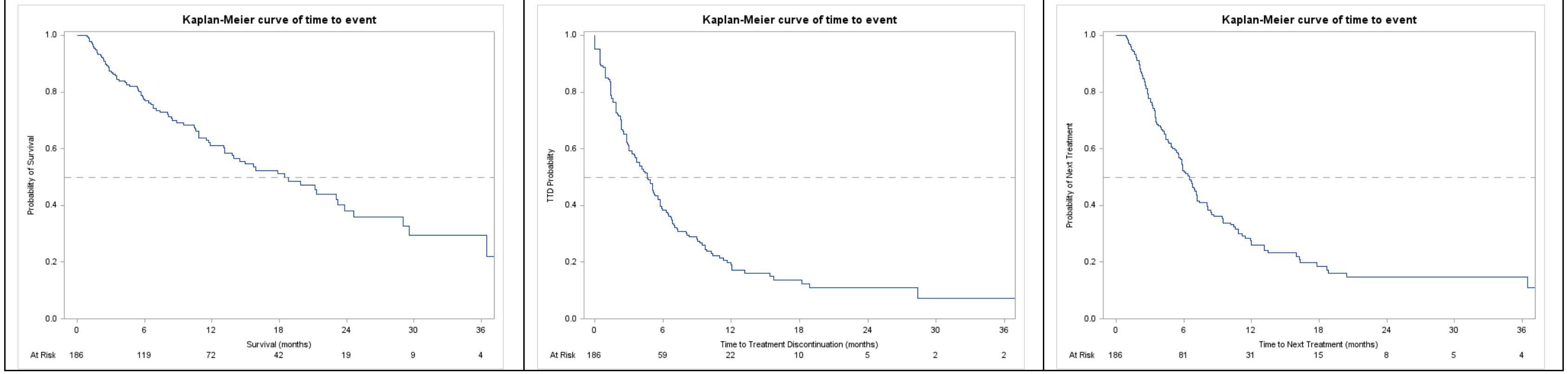

Abbreviations: 1L - first line; 1LM - first-line maintenance; la/mUC - locally advanced or metastatic urothelial carcinoma; PBC, platinum-based chemotherapy

Clinical outcomes were calculated from start of avelumab 1LM

Figure S4. Clinical outcomes among patients with la/mUC initiating 2L EV post avelumab 1LM

A. Overall survival (OS)	B. Real-world time to treatment discontinuation (rwTTD)	C. Real-world time to next treatment (rwTTNT)

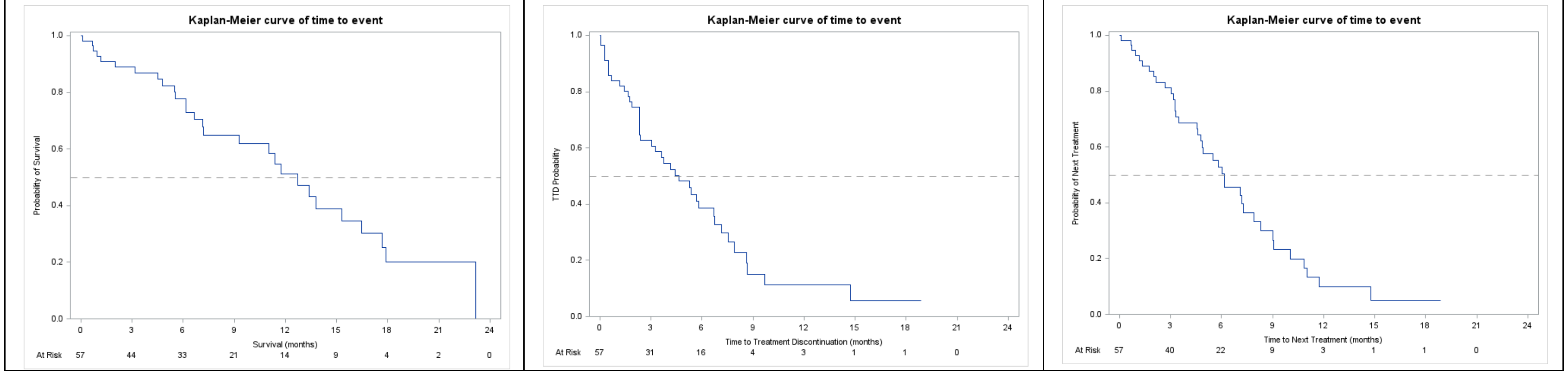

Abbreviation: 2L - second line; 1LM - first-line maintenance; EV - enfortumab vedotin; la/mUC - locally advanced or metastatic urothelial carcinoma

Clinical outcomes were calculated from start of 2L EV
